# EcoBrowser: a web-based tool for visualizing transcriptome data of *Escherichia coli*

**DOI:** 10.1186/1756-0500-4-405

**Published:** 2011-10-13

**Authors:** Xiao Chang, Yun Li, Jie Ping, Xiao-Bin Xing, Han Sun, Peng Jia, Chuan Wang, Yuan-Yuan Li, Yi-Xue Li

**Affiliations:** 1 Bioinformatics Center, Key Lab of Systems Biology, Shanghai Institutes for Biological Sciences, Chinese Academy of Sciences, 320 Yueyang Road, Shanghai 200031, China; 2 Shanghai Center for Bioinformation Technology, 100 Qinzhou Road, Shanghai 200235, China; 3 College of life science and biotechnology, Shanghai Jiaotong University, Shanghai 200120, China; 4 Zilkha Neurogenetic Institute, Department of Psychiatry and Preventive Medicine, University of Southern California, Los Angeles, California 90089, USA

## Abstract

**Background:**

*Escherichia coli *has been extensively studied as a prokaryotic model organism whose whole genome was determined in 1997. However, it is difficult to identify all the gene products involved in diverse functions by using whole genome sequencesalone. The high-resolution transcriptome mapping using tiling arrays has proved effective to improve the annotation of transcript units and discover new transcripts of ncRNAs. While abundant tiling array data have been generated, the lack of appropriate visualization tools to accommodate and integrate multiple sources of data has emerged.

**Findings:**

EcoBrowser is a web-based tool for visualizing genome annotations and transcriptome data of *E. coli*. Important tiling array data of *E. coli *from different experimental platforms are collected and processed for query. An AJAX based genome browser is embedded for visualization. Thus, genome annotations can be compared with transcript profiling and genome occupancy profiling from independent experiments, which will be helpful in discovering new transcripts including novel mRNAs and ncRNAs, generating a detailed description of the transcription unit architecture, further providing clues for investigation of prokaryotic transcriptional regulation that has proved to be far more complex than previously thought.

**Conclusions:**

With the help of EcoBrowser, users can get a systemic view both from the vertical and parallel sides, as well as inspirations for the design of new experiments which will expand our understanding of the regulation mechanism.

## Background

In the past decade, advances on high-throughput sequencing technologies have already made a huge impact on microbiology, providing a fast and economical means of determining whole genome sequences of bacteria [1]. For instance, most of the current completed genome-sequence projects listed on Genomes OnLine Database are microbial. The genome needs to be annotated by identifying the locations and functions of genes. Specifically, the in-depth organizational structure of bacterial genomes still needs to be fully elucidated.

*Escherichia coli *has been widely used as a prokaryotic model organism whose whole genome was sequenced as early as 1997 [2]. The information about its genes, proteins, intergenic regions and biochemical machineries have been collected in the well known databases, including EcoGene, EcoCyc and EcoliWiki [3-5]. However, identifying all the gene products involved in diverse functions has proved difficult to accomplish solely based on whole genome sequences. Thus, microarray data serve as useful complementary information for functional genomics. Some databases are built based on the microarray data like GenExpDB [6]. GenExpDB brings together an extensive collection of gene expression data from the *E.coli *community, so that the gene expression level in different conditions and platforms can be easily compared. Recent advance in biology suggests a wide-spread involvement of noncoding RNA in transcript regulations, but the design of gene microarray can only cover the gene coding regions of the whole genome and many new techniques are aiming to investigate the regulation of no-coding regions. As an unbiased tool to investigate protein binding, gene expression and gene structure on a genome-wide scope, tiling arrays has improved the annotation of transcript units and the discovery of many new transcripts of non-coding and natural antisense RNA [7,8]. While abundant tiling array data have been generated, the lack of appropriate visualization tools to accommodate and integrate multiple sources of data has emerged. The widely used genome browsers such as UCSC genome browser and Ensembl Bacteria reload the entire genome browser page by every action [9,10]. The discontinuous page transitions impair the user's sense of which genomic locus they are viewing and how the displayed data points relate to one another. In addition, as the size of tiling array data is usually very huge, it is also time consuming to upload and display them on the browser server.

We therefore built EcoBrowser which is a web-based visualization tool for searching genome annotations through transcriptome expression profiles of *E.coli*. The major difference between EcoBrowser and GenExpDB is that GeneExpDB focuses on gene expression data. EcoBrowser focuses on visualizing the whole-transcriptome mapping data such as tiling array, therefore the expression level of both coding region and non-coding region can be included and led to further integration analysis. The expression value were transformed into shapes of bule colors for drawing the heatmaps. The heatmap of whole genome were pre-rendered as tiles of images at multiple zoom levels and stored on the server-side. With the help of AJAX technology, a smooth panning and zooming effect can be created by dynamically changing the positional offset of these tiles, fetching new tile images when necessary (without reload the whole page). Thus, genome-wide comparison of expression patterns from independent experiments and genome annotation can be performed by direct comparison which will be helpful in discovering new transcripts, non-coding RNAs and generating a detailed description of the transcription unit architecture. It could also provide clues for further investigation of condition-specific transcriptional regulation.

## Findings

### Methods

The EcoBrowser is composed of a web interface, a database as well as an AJAX based genome browser [11]. The user interface is written in Perl and implemented by using Perl's Common Gateway Interface module (CGI.pm) and Cascaded Style Sheets (CSS). The database stores integrated identified genes and transcription units information obtained from NCBI, EcoCyc and EcoGene [3,4,12]. The transcription unit annotation of *E. coli *is also included according to a recent study [8] . Gene symbole, gene id, transcription unit id and modular unit id can be queried. All the transcriptome datasets about transcriptome analysis were downloaded from Gene Expression Omnibus (GEO). Currently, there are 67 tiling arrays from five publications in EcoBrowser, the description of the data used for the tracks can be found in the "Help" page [8,13-16]. The transcriptome data are displayed by a genome-based heatmap and rendered into a series of images by the statistical language R. In order to make the results from different platforms comparable, we calculte the relatve signal (ranging from 0 to 1) using the following formula:

$$S_{relative}=\frac{S_{i}-\min\left(S\right)}{\max\left(S\right)-\min\left(S\right)}$$

where *S_i _*means the signal value of the ith gene, *S *represents [*S*_1_, *S*_2_,... *S_n_*], where *n *is the number of genes. The shade of blue represents the relative expression level of the probes which continuously cover the entire genome in each track. Jbrowse is to navigate trough the gene and transcription unit predictions [11,17]. The AJAX-based browser offers a faster and smoother navigation through the genome without reloading of the page. The genome annotations are rendered on the client side while the transcriptome expression heatmaps are prerendered and stored on the server.

## Results and Discussion

**Figure 1:**
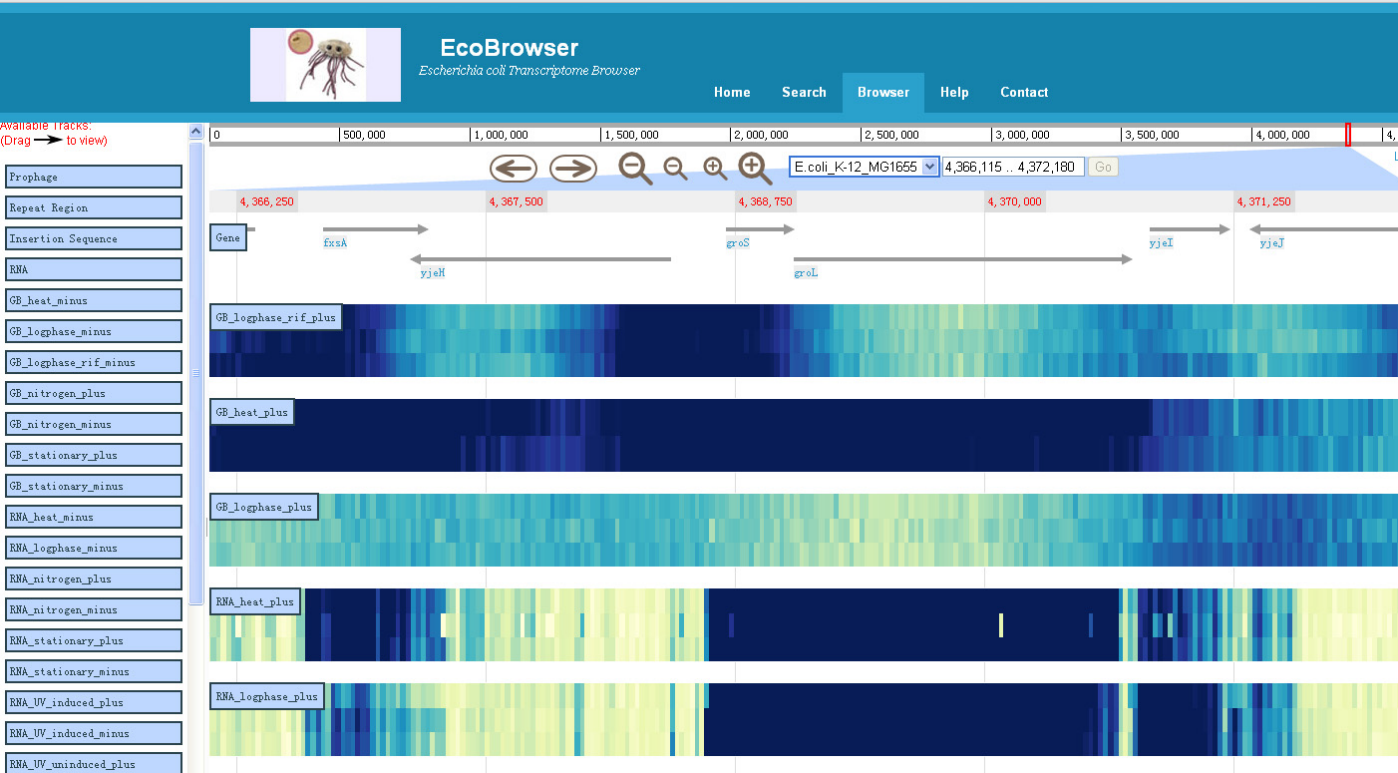
**A snapshot of EcoBrowser**. A snapshot of EcoBrowser displays the gene location and transcriptome data. The tracks in the left panel could be dynamically added and removed by dragging. The shade of blue represents the relative expression level of the probes and the description of the tracks are in the "help" page.

EcoBrowser provides a user-friendly interface. Users can select genomic regions of interest (e.g. via gene or locus IDs) and then select the transcriptome data to be displayed simultaneously on the search page. Taking a well studied heat shock gene, groS (b4142), for example, identified genes or transcription units information is returned by clicking the "Search" button; the list of the optional datasets and annotations shows up by clicking the "display" button. EcoBrowser includes two types of transcriptome analysis data generated by tiling array, transcript expression profiling (like RNA_heat, RNA_logphase) and genome binding/occupancy profiling (like GB_heat, GB_logphase, GB_logphase_rif). Here we choose the datasets including RNA_heat, RNA_logphase, GB_heat, GB_logphase, GB_logphase_rif, and the gene location. More details are on the help page. After clicking the "browse selected button" the selected datasets and annotations will be visualized at the position where the selected gene entry is located (Figure 1). Users can also add or remove tracks to dynamically generate customized views. Hence, a straightforward comparison of the transcriptome data from different sources and under various conditions can be performed.

In the case of groS (b4142) and groL (b4143), the two adjacent genes belonging to the same operon are shown to be co-expressed in the tracks RNA_heat_plus and RNA_logphase_plus. RNA polymerase (RNAP) binds to the gene regions of groS and groL by pulses of heat (GB_heat) while not in the log phase (GB_logphase). The above indicates that firstly the transcription of .groS and groL are activated by the heat pulse; secondly, the transcript of groS and groL are still kept in a high level in the log-phase condition due to their essential role in protein maintenance and cell growth. After combining the static map of Rifampicin-induced RNAP-binding promoter regions (GB_logphase_rif), users can get a better understanding of the process of groS and groL transcription. More findings can be revealed by extending the object to more genes of the whole genome as well as more species.

About 80 of hundreds of predicted sRNAs candidates in silico have been experimentally validated in *E.coli*. However, many more predicted sRNAs located in the intergenic regions shows a high expression levelin EcoBrowser. A recent paper identified 10 new non-coding sRNAs of *E.coli *by using a genome-wide deep-sequencing approach, 9 of them display a clear high expression level in EcoBrowser (details in supplementary, additional file 1) [18]. Thus, biologists can use EcoBrowser as a reference before the experimental validation of a new sRNA candidate. We have collected the predicted sRNA results of *E.coli *from several papers to help users make use of the browser more effectively [19-23]. The prediction information is in "Help" page.

## Conclusions

The EcoBrowser is a valuable tool for researchers. With the help of the integrated genome browser, users can also get a systemic view both from the vertical and parallel sides, as well as inspirations for the design of new experiments which will expand our understanding of the regulation mechanism. Next generation datasets, such as RNA-seq, will also be included in the future when the next generation sequencing technologies have been extensively applied.

## Availability and requirements

Project name: EcoBrowser project

Project home page: http://ecobrowser.biosino.org

Operating systems: Platform independent

Programming language: Javascript, CSS, CGI

Other requirements: None

## Competing interests

The authors declare that they have no competing interests.

## Authors' contributions

YXL and YYL conceived and designed the study. XC, YL, JP, XXB, HS conducted the analyses. XC, YL, CW built the web server. XC, YYL and YXL wrote the manuscript. All authors read and approved the final manuscript.

## Supplementary Material

Additional file 1 **examples of recently reported sRNA display a high expression level in EcoBrowser**. Nine of the ten recently reported sRNA display a high expression level in the according region in EcoBrowser. We selected two of them (one is in forward strand and the other is in reverse strand) as examples. Click here for file
