# Supplementary material for: EcoBrowser: a web-based tool for visualizing transcriptome data of Escherichia coli
Source: BMC Res Notes. 2011 Oct 13;4:405. doi: 10.1186/1756-0500-4-405 (PMC3203075; doi:10.1186/1756-0500-4-405)
Supplement: Additional file 1 — examples of recently reported sRNA display a high expression level in EcoBrowser. Nine of the ten recently reported sRNA display a high expression level in the according region in EcoBrowser. We selected two of them (one is in forward strand and the other is in reverse strand) as examples. [file 1756-0500-4-405-S1.DOC]

*

Nine of the ten reported sRNA display a high expression level in the according region in EcoBrowser. We selected two of them ( one is in forward strand and the other is in reverse strand) as examples.


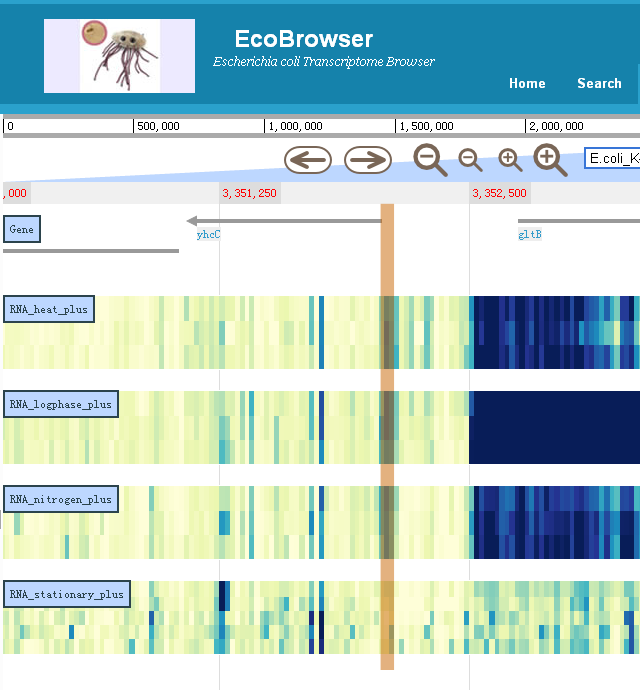


Figure S1. a new sRNAs reported by the recent paper is loacted on the forward strand and next to gene yhcC . The pink marks the according region in EcoBrowser. The track (RNA_heat, RNA_logphase, RNA_nitrogen) shows a high expression level in this non-coding region.


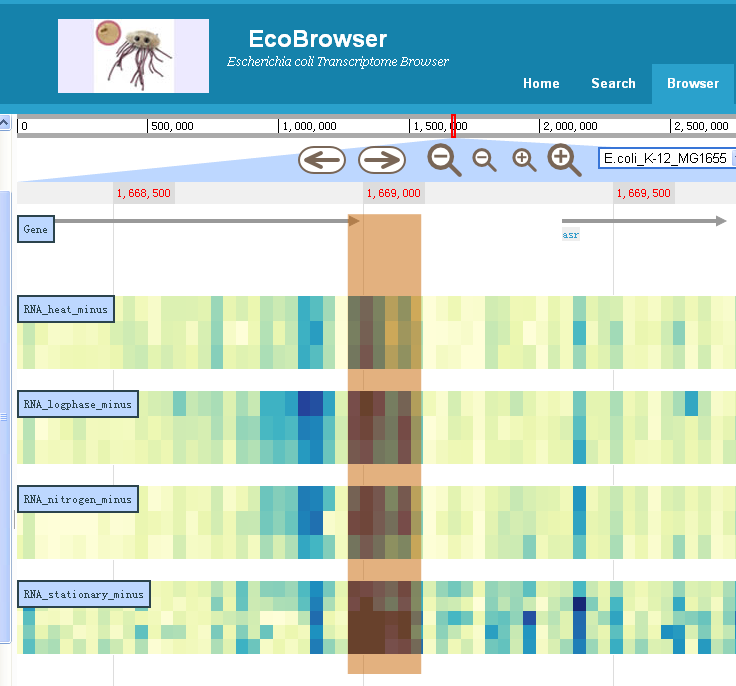


Figure S2. a new sRNAs reported by the recent paper is loacted on the reverse strand and next to gene ynfM ( gene name is not shown on this figure) . The pink marks the according region in EcoBrowser. The track (RNA_heat, RNA_logphase, RNA_nitrogen, RNA_stationary) shows a high expression level in this non-coding region.

Reference

1. Raghavan R, Groisman EA, Ochman H: **Genome-wide detection of novel regulatory RNAs in E. coli**. *Genome Res* 2011.
